# Use of Budesonide in the Treatment of Microscopic Colitis

**DOI:** 10.4103/1319-3767.65188

**Published:** 2010-07

**Authors:** Vikram Tangri, Nilesh Chande

**Affiliations:** Department of Medicine, The University of Western Ontario and London Health Sciences Centre, London, Ontario, Canada

**Keywords:** Budesonide, collagenous colitis, lymphocytic colitis, microscopic colitis

## Abstract

Collagenous colitis and lymphocytic colitis, the two types of microscopic colitis, cause watery diarrhea. Budesonide, a glucocorticoid medication with limited systemic availability, is commonly used to treat these illnesses. Budesonide has proven efficacy in the induction of clinical remission in both collagenous colitis and lymphocytic colitis. Budesonide is effective as a maintenance drug for patients with collagenous colitis, but has not been studied for this indication in patients with lymphocytic colitis. This drug improves quality of life in patients while causing few mild adverse events. Budesonide is an effective treatment of microscopic colitis that is safe and well tolerated.

Microscopic colitis (MC) is a common cause of watery diarrhea, abdominal cramping, and nausea.[1,2] Although endoscopy shows grossly normal colonic mucosa in patients with MC, biopsies demonstrate histological anomalies.[1] The two subtypes of MC are collagenous colitis (CC) and lymphocytic colitis (LC). CC is characterized by a thickened subepithelial collagen deposition on histological analysis, while LC by intraepithelial lymphocytosis.[3] MC is more common in middle aged or elderly patients.[4] Various medications have limited evidence for the treatment of MC, including anti-diarrheal agents such as loperamide, bismuth subsalicylate, mesalamine, cholestyramine, and immunosuppressive therapies.[5,6] Budesonide, a glucocorticoid medication, has been the most widely investigated treatment for both induction and maintenance therapy in patients with MC. This paper summarizes the pharmacology of budesonide, its efficacy, effects on quality of life (QOL), and safety in the treatment of MC.

## PHARMACOLOGY OF BUDESONIDE

Corticosteroids decrease levels of inflammatory cytokines such as interleukin (IL)-1, IL-6, and tumor necrosis factor (TNF)-α through inhibition of protein synthesis and transcription.[7] Unfortunately, systemic corticosteroids are associated with numerous cosmetic and serious adverse events including cushingoid features infection, hypertension, osteoporosis, and diabetes mellitus.[8] Budesonide is a glucocorticoid with extensive first-pass hepatic metabolism by cytochrome P-450 enzymes. Due to limited systemic availability, many of the adverse events associated with systemic corticosteroids are diminished.

## EFFICACY OF BUDESONIDE IN THE TREATMENT OF COLLAGENOUS COLITIS

### Induction of clinical remission

Several randomized controlled trials have demonstrated the efficacy of budesonide in inducing remission in patients with CC. One double-blind placebo-controlled trial involving 28 patients showed decreased stool frequency in 57% patients receiving budesonide (9 mg daily for 8 weeks) compared with 21% in the placebo group. This clinical response was associated with improved stool consistency and a decreased thickness in lamina propria.[9]

A randomized trial of 51 patients showed that clinical remission was significantly higher in patients taking budesonide (9 mg budesonide for 6 weeks) for induction therapy versus those assigned placebo (77% vs. 12%, respectively). Histological improvement was observed in 61% of patients in the budesonide group compared to 5% of patients in the placebo group.[10]

Another placebo-controlled trial involving 20 patients randomized to budesonide therapy (9 mg for 4 weeks, 6 mg for 2 weeks, and 3 mg for 2 weeks) reported 100% subjects of the treatment group achieving a clinical response compared to 20% in the placebo group. There were also reductions in stool weight and frequency, along with reduced collagen layer thickness on biopsy in those taking budesonide.[11]

More recent trials of budesonide, as maintenance therapy, have also shown benefit of this drug in achieving remission for patients with CC in their open-label induction arms. After 6 weeks of therapy with 9 mg of budesonide daily, 46 of 48 patients (96%) achieved clinical remission in one study.[12] Another study achieved remission in 34 of 42 patients (81%) with CC taking budesonide 9 mg daily for six weeks.[13] Thus, several studies support the use of budesonide for the induction of remission in patients with CC, usually involving 6-9 mg of budesonide daily for 6-8 weeks duration.

### Maintenance therapy

Although efficacious in inducing remission, many patients on budesonide therapy experience relapse after cessation of the drug. One study reported a relapse in 8 of 10 patients with CC within 8 weeks of stopping budesonide.[11] Another reported a cumulative relapse rate of 61% in 16 months of follow up for such patients, with age less than 60 years being significantly correlated with relapse.[14]

Two recent randomized controlled trials have evaluated the use of budesonide (6 mg daily) for maintaining clinical remission in patients with CC. One study (*n*=46) found that patients randomized to budesonide maintenance therapy had a significantly higher rate of remission maintenance when compared to those randomized to placebo therapy (74% vs. 35%, respectively) over a 6 month period of time post-induction. Most relapses occurred in the first 2 months after induction.[12] Another trial of 34 patients demonstrated similar results, with higher rates of clinical remission maintained on 24 weeks of budesonide therapy compared to placebo (77% vs. 12%, respectively). However, budesonide may not alter the natural course of CC, since the relapse rate after 24 weeks of budesonide therapy was similar to the relapse rate after 6 weeks of induction therapy.[13] A recent systematic review had shown efficacy of budesonide for both induction and maintenance therapy for CC.[6]

## EFFICACY OF BUDESONIDE IN THE TREATMENT OF LYMPHOCYTIC COLITIS

Relatively few studies have been published on the use of budesonide in LC. One retrospective analysis showed that four of seven patients with LC experienced clinical response (57%) with budesonide treatment.[15] A Swedish multicenter retrospective study demonstrated that 14 of 17 patients (82%) with LC treated with budesonide showed some treatment response.[6] Only one randomized controlled trial (*n*=42) of budesonide therapy for LC has been published, finding that 86% of patients with LC randomized to induction therapy budesonide (9 mg daily) achieved clinical response at 6 weeks compared to 48% assigned placebo.[16] Budesonide therapy has also been found to induce higher rates of histological remission compared with placebo.[6,16] Thus, budesonide therapy holds promise in the treatment of LC, although no trials of budesonide for maintenance of remission have been published.

## BUDESONIDE AND QUALITY OF LIFE

Budesonide induction therapy improves QOL in patients with CC, including symptoms, emotional functioning, and physical functioning measured by the gastrointestinal quality of life index (GIQLI).[17] Other areas that budesonide has been found to improve in patients with CC include daily activities and toileting.[12] Budesonide for LC has not shown to be robust in effect, as one study reported improvement in the physical sum score QOL in both budesonide and placebo groups; however, mean mental sum score was unchanged in these groups.[16]

## SAFETY AND ADVERSE EVENTS

Budesonide has relatively few side effects or safety concerns, due to its extensive metabolism by hepatic cytochrome P-450 enzymes. Most studies report mild adverse events that are transient in nature; more common side effects include headache, nausea, and dizziness.[9,10,13,16] In a study of budesonide for maintenance therapy for patients with CC, 89% of patients rated the tolerability of the drug as “very good” or “good” after 6 weeks of treatment; however, only 61% of patients reported the same tolerability ratings after 6 months of treatment.[11] The risk of osteoporosis due to prolonged steroid use is a concern. However, budesonide has limited systemic availability and there remains little scientific data in this regard. Overall, budesonide has been shown to be safe with minimal mild adverse events, when used for a short-term course of treatment.

## CONCLUSION

Budesonide is a glucocorticoid medication commonly used to treat both CC and LC. Although most studies have focused on treatment of CC, similar benefits have been noted in patients with LC. It is an effective medication for inducing clinical remission with doses of 6 mg to 9 mg daily for 6 to 8 weeks in both CC and LC. Budesonide is also an effective drug in maintaining remission in CC. This medication has been shown to improve quality of life in patients, while causing few mild adverse events. Thus, budesonide is an efficacious and well-tolerated treatment of microscopic colitis.
